# Comparative Cardiovascular Outcomes in Acalabrutinib vs. Zanubrutinib in B-Cell Malignancies: A TriNetX Data Study

**DOI:** 10.3390/curroncol33100641

**Published:** 2026-10-21

**Authors:** Maan Awad, Sunanda Tah, Syed Ahmad, Het Patel, Prajwal Kumsi Sreedhar, Shanmukh Sai Pavan Lingamsetty, Abdulrahman Murshid, Brijesh Patel

**Affiliations:** 1 Department of Cardiology, West Virginia University Heart and Vascular Institute, Morgantown, WV 26506, USA; 2 Department of Medicine, West Virginia University, Parkersburg, WV 26104, USA; sunanda.tah@hsc.wvu.edu; 3 Department of Medicine, West Virginia University, Morgantown, WV 26506, USAabdulrahman.murshid@hsc.wvu.edu (A.M.); 4 Department of Medicine, Willis Knighton Health System, Shreveport, LA 71103, USA; patel@wkhs.com (H.P.); pkumsisre@wkhs.com (P.K.S.); 5 Department of Internal Medicine, Mamata Medical College, Khammam 507002, Telangana, India; shanmukh.lingamsety@stvincenthospital.com; 6 Department of Internal Medicine, Division of Cardiology, Indiana University, Indianapolis, IN 46202, USA; bpatel9@iuhealth.org

**Keywords:** cardiotoxicity, tyrosine kinase inhibitors, acalabrutinib, zanubrutinib

## Abstract

Next-generation Bruton’s tyrosine kinase inhibitors (BTKis) such as acalabrutinib and zanubrutinib have improved treatment outcomes in B-cell malignancies, including chronic lymphocytic leukemia (CLL), mantle cell lymphoma (MCL), and Waldenström macroglobulinemia (WM), while reducing off-target toxicities compared to ibrutinib. However, their comparative cardiovascular safety profiles remain poorly characterized. This study evaluates real-world cardiovascular and clinical outcomes between zanubrutinib and acalabrutinib using a large multi-center database. We conducted a retrospective cohort study using the TriNetX Research Network (October 2017–December 2024) including adult patients with CLL, MCL, WM, or small B-cell lymphoma treated with zanubrutinib or acalabrutinib. Patients with prior exposure to other BTKis were excluded. After 1:1 propensity score matching, 2887 patients were included in each cohort. We assessed one-year outcomes including new-onset atrial fibrillation/flutter, cerebral infarction, hypertension, and all-cause mortality using Cox proportional hazards modeling. The rate of AF/flutter events observed in the zanubrutinib group (4.05%) was numerically lower compared to the acalabrutinib group (5.25%). Time-to-event analysis did not demonstrate a statistically significant difference in AF risk over time (HR 0.777, 95% CI 0.597–1.012; *p* = 0.06), which was similar in binary analysis (OR 0.761, 95% CI 0.581–0.997; *p* = 0.05). Cerebral infarction rates were similar between groups (1.09% vs. 1.12%), with no significant difference in time-to-event analysis (HR 0.984, 95% CI 0.596–1.626; *p* = 0.95) or binary analysis (*p* = 0.91). There was no significant difference in onset of hypertension in the zanubrutinib cohort (HR 1.199, 95% CI 0.957–1.501; *p* = 0.11) All-cause mortality was not statistically significant between the two groups (HR 0.865, 95% CI 0.740–1.012; *p* = 0.07). In this large, real-world analysis, fewer AF/flutter events were observed with zanubrutinib at one year; however, time-to-event analysis did not demonstrate a statistically significant difference in AF risk over time. Notably, hypertension, cerebral infarction, and all-cause mortality occurred at similar rates. This suggests zanubrutinib and acalabrutinib share similar cardiovascular safety profiles overall. However these findings are hypothesis-generating and require prospective validation before definitive conclusions about cardiovascular risk can be drawn.

## Simple Summary

Next-generation Bruton’s tyrosine kinase inhibitors (BTKis) such as acalabrutinib and zanubrutinib have improved treatment outcomes in B-cell malignancies, all while reducing side effects of off-target toxicities when compared to ibrutinb. However, their cardiovascular side effects remain uncertain. We created a retrospective cohort study using TriNetX US Collaborative Network (TriNetX, LLC, Cambridge, MA, USA) (2017–2024), which included adults with chronic lymphocytic leukemia (CLL), Waldenstrom macroglobulinemia (WM), mantle cell lymphoma (MCL), or small B-cell lymphoma, treated with zanubrutinib or acalabrutinib. Propensity scoring was done, which resulted in 2887 patients in each cohort. One-year cardiovascular outcomes and mortality were assessed. Zanubrutinib showed fewer atrial fibrillation/flutter events (4.05% vs. 5.25%); however, the difference between the groups was not statistically significant (*p* = 0.06). The incidence of hypertension, stroke, and all-cause mortality were similar between the groups. Overall, zanubrutinib and acalabrutinib showed comparable cardiovascular safety profiles. These findings should only be used to guide further research as future prospective trials are needed before conclusions on cardiovascular safety can be made.

## 1. Introduction

Bruton’s tyrosine kinase inhibitors (BTKis) changed the management of B-cell malignancies such as mantle cell lymphoma (MCL), Waldenstrom macroglobulinemia (WM), and chronic lymphocyte leukemia (CLL), because of their safety profile and better therapeutic effect. Comparatively, ibrutinib, a first generation BTKi, is effective but has a high-risk profile and some of the side effects include: atrial fibrillation (AF), hypertension, heart failure, and ventricular arrhythmias [1].

In response, next-generation BTKis—acalabrutinib and zanubrutinib—were developed to enhance target specificity and reduce off-target adverse events. Retrospective single-center studies have shown that acalabrutinib is associated with an increased incidence of AF, atrial enlargement, ventricular arrhythmias, and early development of hypertension [2,3]. Few phase III trials have reported an increased incidence of AF and hypertension with zanubrutinib [4,5], but its impact on heart failure, ventricular arrhythmias, and sudden cardiac death remains unclear [6].

While both agents have shown favorable safety profiles compared to ibrutinib in clinical trials, cardiovascular risk may not be entirely mitigated. Most studies compare next-generation BTKis to ibrutinib [7]; however, direct comparisons between acalabrutinib and zanubrutinib are limited. This study aims to compare the cardiovascular and all-cause mortality outcomes of zanubrutinib and acalabrutinib use in a real-world population to ascertain any differences that may influence clinical use of these medications.

## 2. Materials and Methods

This retrospective cohort study utilized the TriNetX US Collaborative Network (TriNetX, LLC, Cambridge, MA, USA), accessed on 16 November 2025. TriNetX aggregates de-identified electronic medical records from 104 healthcare organizations, encompassing 140 million U.S. patients. The study period extended from 1 October 2017 to 31 December 2024. As the study used only de-identified data, it was exempt from institutional review board (IRB) oversight.

Adults aged 18 years or older with ICD-10-coded diagnoses of CLL, MCL, WM, or small B-cell lymphoma were included. Patients were assigned to one of two mutually exclusive cohorts based on BTKi exposure; those receiving zanubrutinib without prior exposure to ibrutinib, acalabrutinib, or pirtobrutinib, and those receiving acalabrutinib without exposure to other BTKis. Patients with a history of using more than one BTKi were excluded from the analysis.

To control for confounding, 1:1 propensity score matching was performed using a nearest-neighbor algorithm with a 0.2 standard deviation caliper. Matching variables included demographic factors (age, sex, race, ethnicity), cardiovascular comorbidities (ischemic heart disease, heart failure, cerebrovascular disease), other comorbidities (diabetes, chronic kidney disease, liver cirrhosis, hyperthyroidism, HIV, chronic pulmonary disease, sleep apnea, obesity), concurrent therapies (corticosteroids, anticoagulants such as warfarin, apixaban, rivaroxaban, chemotherapeutic agents such as anthracyclines, trastuzumab, tyrosine kinase inhibitors and others), and relevant laboratory and clinical parameters including renal function, hepatic function, serum albumin, and Eastern Cooperative Oncology Group [ECOG] performance status.

Primary outcomes evaluated over a one-year follow up period included new-onset atrial fibrillation or flutter, cerebral infarction, incident hypertension, and all-cause mortality. Baseline characteristics were assessed using standardized mean differences. Odds ratios (ORs) with 95% confidence intervals (CIs) were calculated for all outcomes. Time-to-event data were analyzed using Cox proportional hazards modeling and Kaplan–Meier survival curves with log-rank tests. The proportional hazards assumption was evaluated using Schoenfeld residual analysis for all outcomes. The primary analytic time window was set at one year, as BTKi-related cardiovascular effects are expected to appear predominantly within this period, and medication adherence and treatment duration beyond one year are not captured.

The index event was set as the first recorded prescription for zanubrutinib or acalabrutinib within the study period. Outcomes were established using ICD-10 codes. New-onset atrial fibrillation (AF) or flutter was identified by ICD-10 codes in Trinetx (I48.0–I48.92). It is important to note that patients with a pre-existing diagnosis of AF or flutter before the index date were excluded. Similarly, cerebral infarction was identified using codes I63.0–163.9, and patients with any previous history of stroke before the index event were excluded. Hypertension was identified using codes I110-I116.9; again, any previous history of hypertension was excluded. For all-cause mortality Trinetx uses vital status records, ICD-10 codes to aggregate that information into the database.

## 3. Results

Following propensity score matching, each cohort comprised 2887 patients. Prior to matching, the zanubrutinib group had a higher prevalence of apixaban use and hypothyroidism, while the acalabrutinib group had a higher prevalence of Black or African American race and bortezomib use. The mean age was 72.1 years in the zanubrutinib group and 72.0 years in the acalabrutinib group (*p* = 0.88). The proportion of male patients was similar between cohorts (62.9% for zanubrutinib vs. 62.5% for acalabrutinib; *p* = 0.79). Comorbid conditions were well balanced between groups after matching (Table 1), except for serum albumin, which remained marginally imbalanced after matching (mean 3.9 vs. 4.0 g/dL; standardized difference 0.197), and was retained as a covariate in sensitivity analyses.

**Figure 1:**
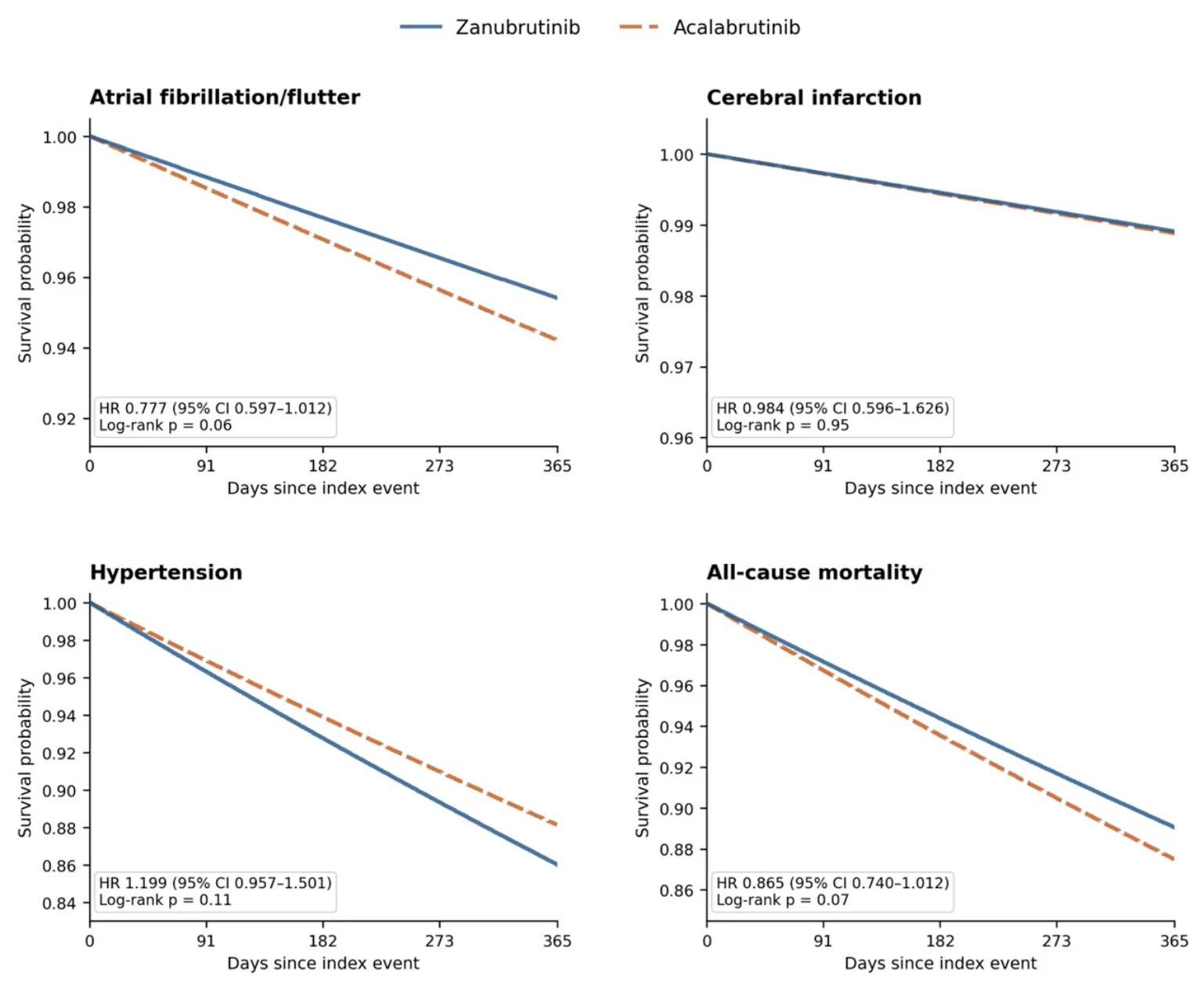
Kaplan–Meier survival curves by outcome. Curves are reconstructed from reported one-year survival probabilities and log-rank statistics for illustrative purposes; they approximate but are not digitized from patient-level TriNetX survival data. Log-rank *p*-values are shown for each outcome.

**Figure 2:**
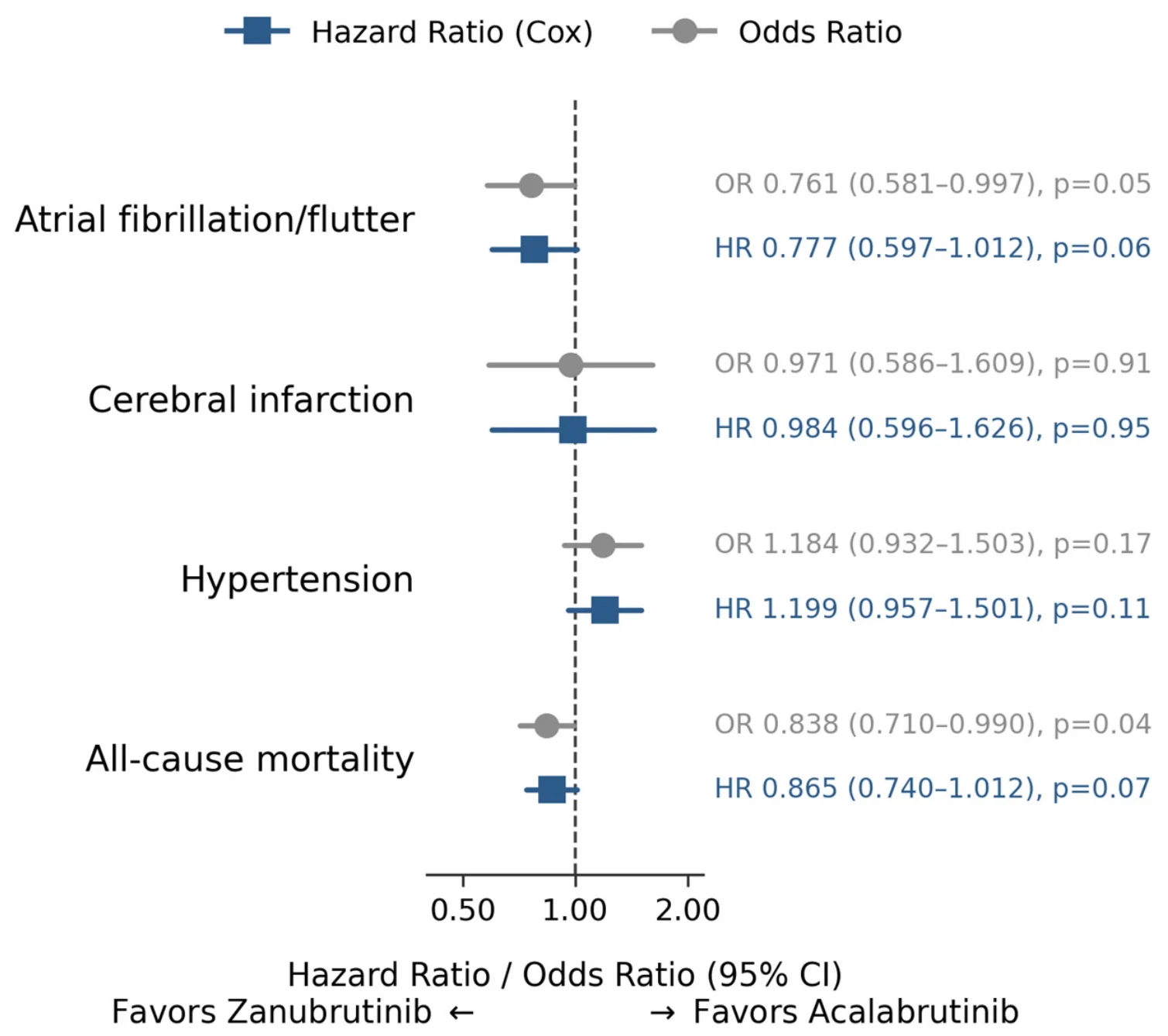
Forest plot of hazard ratios and odds ratios for primary outcomes. Squares represent hazard ratios (Cox proportional hazards models); circles represent odds ratios. Horizontal lines represent 95% confidence intervals. The dashed vertical line indicates the line of no effect (HR/OR = 1.0). HR is presented as the primary time-to-event metric; OR is presented as a corroborating binary measure.

Fewer events of atrial fibrillation or flutter were observed in the zanubrutinib cohort (4.05%, 98/2421) compared to the acalabrutinib cohort (5.25%, 126/2398; Table 2). Time-to-event analysis did not demonstrate a statistically significant difference in AF risk over time (HR 0.777, 95% CI 0.597–1.012; *p* = 0.06; Figure 1), and the proportional hazards assumption was valid (Schoenfeld residuals *p* = 0.24). Binary analysis was directionally consistent, with fewer events in the zanubrutinib group (OR 0.761, 95% CI 0.581–0.997; *p* = 0.05; Figure 2).

Cerebral infarction rates were similar between groups at one year (1.09% for zanubrutinib, 30/2761 vs. 1.12% for acalabrutinib, 31/2772). Time-to-event analysis showed no significant difference in time to cerebral infarction (HR 0.984, 95% CI 0.596–1.626; *p* = 0.95; Figure 1), and binary analysis was concordant (OR 0.971, 95% CI 0.586–1.609; *p* = 0.91; Figure 2).

Time-to-event analysis for onset of hypertension showed no significant difference between the two groups (HR 1.199, 95% CI 0.957–1.501; *p* = 0.11; Figure 1), and the proportional hazards assumption was valid (Schoenfeld residuals *p* = 0.64). The rate of hypertension onset was similar between groups (12.3% zanubrutinib, 165/1344 vs. 10.6% acalabrutinib, 140/1324; OR 1.184, 95% CI 0.932–1.503; *p* = 0.17; Figure 2). While neither reached statistical significance, this directional finding is clinically relevant given the recognized propensity of BTKis to induce hypertension within weeks of initiation. The one-year hypertension-free survival was 86.01% for zanubrutinib versus 88.13% for acalabrutinib.

All-cause mortality at one year was numerically lower in the zanubrutinib group (10.04%, 289/2878) compared to the acalabrutinib group (11.75%, 338/2876). Cox proportional hazards analysis showed a numerically lower HR with zanubrutinib that did not reach statistical significance (HR 0.865, 95% CI 0.740–1.012; *p* = 0.07; Figure 1), and the proportional hazards assumption was valid (Schoenfeld residuals *p* = 0.83). Binary analysis was directionally consistent (OR 0.838, 95% CI 0.710–0.990; *p* = 0.04; Figure 2), though it reached statistical significance; however, given the primacy of the time-to-event analysis, these findings should be interpreted cautiously. The one-year survival probability was 89.04% for zanubrutinib versus 87.47% for acalabrutinib.

E-value analysis was also done (Table 3). The E-value for the observed association between zanubrutinib and atrial fibrillation was around 1.89, with an E-value of 1.06 for the confidence limit closest to the null. This means that unmeasured confounding could have accounted for the borderline association. The E-values for cerebral infarction, all-cause mortality, hypertension were 1.14, 1.58, and 1.69. The confidence intervals included the null for these outcomes.

## 4. Discussion

In this one-year real-world analysis, fewer AF/flutter events were observed with zanubrutinib; however, time-to-event analyses did not reach statistical significance for the outcome, suggesting no clear difference in longitudinal risk between agents. Notably, hypertension, cerebral infarction, and all-cause mortality occurred at similar overall rates between the zanubrutinib and acalabrutinib cohorts. These findings suggest that second-generation BTKis may exhibit similar cardiovascular risk profiles.

Arrhythmias caused by BTKi tend to happen because of off-target kinase inhibition. This inhibition can happen by interleukin-2-inducible T-cell kinase (ITK), bone marrow-expressed kinase (BMX), tyrosine kinases expressed in hepatocellular carcinoma (TEC) [8,9,10]. Acalabrutinib has demonstrated higher BTK selectivity and limited activity against off-target kinases such as ITK and TEC [11]. Zanubrutinib similarly avoids off-target inhibition and achieves high BTK occupancy in lymphoid tissue [1].

Previous literature has been limited in comparing these agents. There was an indirect analysis which showed no significant difference in AF risk between these drugs [5]. There were fewer instances of AF reported in the zanubrutinib group; however, when the time-to-event analysis was looked at, it did not show a statistically significant difference in AF risk over time (HR 0.777; *p* = 0.06). The divergence between OR and HR for AF likely reflects differences in cumulative event counts versus temporal distribution of events, with Cox modeling indicating no consistent time-dependent risk difference within one year; these results suggest a hypothesis-generating signal rather than a definitive understanding of AF risk. The one-year analytic window was chosen as BTKi-related cardiovascular effects including AF are expected to manifest predominantly within this period, and medication use would be highest in this timeframe.

The absence of a significant difference in cerebral infarction at one year (*p* = 0.95) was consistent across both the Cox HR and OR analyses. Prior mechanistic data suggest zanubrutinib may exert more favorable effects on endothelial function and platelet aggregation than other BTKis, possibly reducing thromboembolic risk beyond the risk posed by AF alone, our one-year data do not support a significant difference in stroke risk. Larger studies with longer follow-up may be required to detect such an effect if present.

Time-to-event analysis showed a trend toward earlier onset of hypertension in the zanubrutinib cohort (*p* = 0.11), while overall rates were similar between groups (12.3% vs. 10.6%; *p* = 0.17). This finding, while not statistically significant, is directionally consistent and clinically relevant: BTKi-associated hypertension is recognized to arise within weeks of treatment initiation, and even a non-significant trend toward earlier onset may carry implications for blood pressure monitoring and management. This finding aligns with prior comparative data. An indirect comparison found that acalabrutinib monotherapy was associated with significantly lower odds of any grade of hypertension compared to zanubrutinib (OR 0.44; 95% CI 0.20–0.99) [5]. Similarly, another study reported a lower risk of new-onset hypertension with acalabrutinib compared to ibrutinib (16.3% vs. 27.7%) [7]. Our data suggests that over time, the difference in hypertension between the two BTKis may be similar overall.

For all-cause mortality, Cox proportional hazards analysis showed a numerically lower hazard with zanubrutinib that did not reach statistical significance (HR 0.865, 95% CI 0.740–1.012; *p* = 0.07), indicating no clear difference in longitudinal mortality risk between the two agents. The OR was statistically significant (OR 0.838; *p* = 0.04); however, the HR was deemed to be foremost finding given the importance of measuring death over time. Regardless, we do recommend further investigation in future studies given the directionality of both findings (i.e., HR < 1.00 and *p* < 0.10). In conclusion, the results from this study are consistent with recent meta-analysis of BTK inhibitor therapy in high-risk relapsed/refractory CLL, which showed that zanubrutinib had improved progression free survival when compared to acalabrutinib. However overall survival did not reach statistical significance [12]. Overall, these findings are hypothesis generating and require prospective trials before mortality benefit can be concluded.

### Limitations

Limitations for this study were directly related to its retrospective design and the use of electronic health records (EHRs), for data collection. Although propensity score matching was used to limit confounding, there is residual confounding from unmeasured variables (disease severity, socioeconomic factors), which could not be differentiated. Secondly, using ICD codes can cause misclassification bias because it is hard to prove how accurately the coding was done initially, and there are no checks and balances with that. The third limitation was that TriNetX does not have detailed information on medication adherence or treatment duration, and both of those factors could affect cardiovascular outcomes. Fourth, the timing of outcome onset relative to BTKi initiation may not be precisely captured, and temporal relationships should therefore be interpreted with caution. Fifth, cause-specific mortality and cancer-specific progression data were not available, limiting our ability to attribute mortality directly to cardiovascular or oncologic events. Sixth, as TriNetX is a federated, continuously updated database, the underlying patient records may change over time as participating institutions add or revise EHR data; all results presented here reflect the database state at the time of access on 16 November 2025, and cohort sizes may differ if the analysis were repeated at a later date.

We also had a one-year follow-up period to capture immediate or subacute cardiovascular complications related to treatment. However, in our database we could not account for treatment intolerance or discontinuation. There is no filter for adherence to treatment. Finally, while the large, real-world dataset enhances generalizability, the findings require confirmation through prospective, randomized head-to-head trials.

## 5. Conclusions

In this large, real-world analysis using one-year follow-up, fewer AF/flutter events were observed with zanubrutinib compared to acalabrutinib in patients with B-cell malignancies; however, time-to-event analysis did not demonstrate a statistically significant difference in AF risk over time. No clear difference in mortality risk over time was identified, though there was a possible trend towards decreased mortality with zanubrutinib. Hypertension and cerebral infarctions occurred at similar overall rates between the groups. These findings are hypothesis-generating and require prospective validation.

## Figures and Tables

**Table 1:** Baseline characteristics after 1:1 propensity score matching. Data are derived from the TriNetX Research Network output (accessed 16 November 2025).

Characteristic	Zanubrutinib (*n* = 2887)	Acalabrutinib (*n* = 2887)	Std. Diff. (SMD)
**Demographics**			
Age at index, years (mean ± SD)	72.1 ± 10.3	72.0 ± 10.4	0.004
Male sex, *n* (%)	1815 (62.9%)	1805 (62.5%)	0.007
Female sex, *n* (%)	1072 (37.1%)	1082 (37.5%)	0.007
White race, *n* (%)	2380 (82.4%)	2383 (82.5%)	0.003
Black or African American race, *n* (%)	188 (6.5%)	174 (6.0%)	0.020
Asian race, *n* (%)	58 (2.0%)	58 (2.0%)	<0.001
Race not reported, *n* (%)	187 (6.5%)	190 (6.6%)	0.004
Hispanic or Latino ethnicity, *n* (%)	81 (2.8%)	80 (2.8%)	0.002
Not Hispanic or Latino, *n* (%)	2448 (84.8%)	2474 (85.7%)	0.025
Ethnicity not reported, *n* (%)	358 (12.4%)	333 (11.5%)	0.027
**Comorbidities**			
Ischemic heart disease, *n* (%)	708 (24.5%)	682 (23.6%)	0.021
Heart failure, *n* (%)	366 (12.7%)	336 (11.6%)	0.032
Sleep apnea, *n* (%)	392 (13.6%)	380 (13.2%)	0.012
Diabetes mellitus, *n* (%)	572 (19.8%)	555 (19.2%)	0.015
Overweight and obesity, *n* (%)	492 (17.0%)	453 (15.7%)	0.037
Chronic kidney disease, *n* (%)	539 (18.7%)	492 (17.0%)	0.043
Chronic lower respiratory disease, *n* (%)	677 (23.4%)	670 (23.2%)	0.006
Other hypothyroidism, *n* (%)	491 (17.0%)	485 (16.8%)	0.006
Thyrotoxicosis/hyperthyroidism, *n* (%)	46 (1.6%)	50 (1.7%)	0.011
Fibrosis/cirrhosis of liver, *n* (%)	49 (1.7%)	50 (1.7%)	0.003
HIV disease, *n* (%)	10 (0.3%)	10 (0.3%)	<0.001
Long-term steroid use, *n* (%)	180 (6.2%)	178 (6.2%)	0.003
**Concurrent Medications**			
Warfarin, *n* (%)	142 (4.9%)	143 (5.0%)	0.002
Apixaban, *n* (%)	389 (13.5%)	384 (13.3%)	0.005
Rivaroxaban, *n* (%)	159 (5.5%)	143 (5.0%)	0.025
Doxorubicin, *n* (%)	73 (2.5%)	76 (2.6%)	0.007
Cyclophosphamide, *n* (%)	124 (4.3%)	116 (4.0%)	0.014
Fluorouracil, *n* (%)	136 (4.7%)	121 (4.2%)	0.025
Bortezomib, *n* (%)	33 (1.1%)	26 (0.9%)	0.024
Bevacizumab, *n* (%)	16 (0.6%)	17 (0.6%)	0.005
**Laboratory Parameters**			
Serum creatinine, mg/dL (mean ± SD)	1.3 ± 5.7	1.7 ± 9.1	0.045
Total bilirubin, mg/dL (mean ± SD)	0.7 ± 0.5	0.7 ± 0.7	0.004
Serum albumin, g/dL (mean ± SD)	3.9 ± 0.6	4.0 ± 0.6	0.197 *
AST, U/L (mean ± SD)	25.8 ± 20.8	26.9 ± 43.7	0.032
ALT, U/L (mean ± SD)	21.8 ± 23.6	23.0 ± 50.7	0.031
ECOG performance status (mean ± SD)	0.7 ± 1.0	0.7 ± 0.8	0.052

Abbreviations: SMD, standardized mean difference; SD, standard deviation; AST, aspartate aminotransferase; ALT, alanine aminotransferase; ECOG, Eastern Cooperative Oncology Group; HIV, human immunodeficiency virus. SMD < 0.1 generally indicates adequate balance between cohorts. * Serum albumin remained marginally imbalanced after matching (SMD 0.197) and was retained as a covariate in sensitivity analyses. Variables not individually tabulated here (e.g., additional chemotherapeutic and biologic agents matched in the propensity score model) were balanced between groups (SMD < 0.1).

**Table 2:** Primary outcomes at one-year follow-up.

Outcome	Zanubrutinib *n* (%)	Acalabrutinib *n* (%)	HR (95% CI)	OR (95% CI)
Atrial fibrillation/flutter	98 (4.05)	126 (5.25)	0.777 (0.597–1.012); *p* = 0.06	0.761 (0.581–0.997); *p* = 0.05
Cerebral infarction	30 (1.09)	31 (1.12)	0.984 (0.596–1.626); *p* = 0.95	0.971 (0.586–1.609); *p* = 0.91
Hypertension	165 (12.3)	140 (10.6)	1.199 (0.957–1.501); *p* = 0.11	1.184 (0.932–1.503); *p* = 0.17
All-cause mortality	289 (10.04)	338 (11.75)	0.865 (0.740–1.012); *p* = 0.07	0.838 (0.710–0.990); *p* = 0.04

Abbreviations: HR, hazard ratio; OR, odds ratio; CI, confidence interval. Cox proportional hazards model; the proportional hazards assumption was met for all outcomes via Schoenfeld residual analysis. Hypertension reported among patients without pre-existing hypertension at baseline (*n* = 1344 zanubrutinib; *n* = 1324 acalabrutinib).

**Table 3:** E-value analysis table.

Outcome	E-Value (Point Estimate)	E-Value (CI)
Atrial Fibrillation	1.89	1.06
Cerebral Infarction	1.14	1.00
Hypertension	1.69	1.00
All-Cause Mortality	1.58	1.00

E-values for the confidence interval are calculated to the closet null. Value greater than 1.00 shows that the confidence interval includes the null.

## Data Availability

Data is publicly available as mentioned in the Methods section.

## References

[1] Tam C.S., Trotman J., Opat S., Burger J.A., Cull G., Gottlieb D., Harrup R., Johnston P.B., Marlton P., Munoz J. (2019). Phase 1 study of the selective BTK inhibitor zanubrutinib in B-cell malignancies and safety and efficacy evaluation in CLL. Blood.

[2] Azali L., Hazelden L., Wiczer T., Palettas M., Thomas R., Aossey C., Blachly J.S., Grever M.R., Kittai A.S., Rogers K.A. (2020). Evaluation of the Incidence and Risk Factors Associated with Major Cardiovascular Events in Patients Receiving Acalabrutinib Therapy. Blood.

[3] Chen S.T., Azali L., Rosen L., Zhao Q., Wiczer T., Palettas M., Gambril J., Kola-Kehinde O., Ruz P., Rogers K. (2022). Incident hypertension and clinical outcomes after acalabrutinib initiation for B-cell malignancies. J. Am. Coll. Cardiol..

[4] Brown J.R., Eichhorst B., Hillmen P., Jurczak W., Kaźmierczak M., Lamanna N., O’Brien S.M., Tam C.S., Qiu L., Zhou K. (2023). Zanubrutinib or ibrutinib in relapsed or refractory chronic lymphocytic leukemia. N. Engl. J. Med..

[5] Kittai A.S., Allan J.N., James D., Bridge H., Miranda M., Yong A.S.M., Fam F., Roos J., Shetty V., Skarbnik A. (2024). An indirect comparison of acalabrutinib with and without obinutuzumab vs zanubrutinib in treatment-naive CLL. Blood Adv..

[6] Quartermaine C., Ghazi S.M., Yasin A., Awan F.T., Fradley M., Wiczer T., Kalathoor S., Ferdousi M., Krishan S., Habib A. (2023). Cardiovascular Toxicities of BTK Inhibitors in Chronic Lymphocytic Leukemia: *JACC: CardioOncology* State-of-the-Art Review. Cardio Oncol..

[7] Nunes R.A.B., Avezum Á., de Oliveira Marques M., Baiocchi O.C.C.G., Bachour P. (2024). Three-year cardiovascular and non-cardiovascular adverse events in patients with chronic lymphocytic leukemia or small cell lymphocytic lymphoma treated with Bruton tyrosine kinase inhibitors acalabrutinib or ibrutinib: A real-world analysis. Ann. Hematol..

[8] Byrd J.C., Brown J.R., O’Brien S., Barrientos J.C., Kay N.E., Reddy N.M., Coutre S., Tam C.S., Mulligan S.P., Jaeger U. (2014). Ibrutinib versus ofatumumab in previously treated chronic lymphoid leukemia. N. Engl. J. Med..

[9] Estupiñán H.Y., Berglöf A., Zain R., Smith C.I.E. (2021). Comparative analysis of BTK inhibitors and mechanisms underlying adverse effects. Front. Cell Dev. Biol..

[10] Ganatra S., Sharma A., Shah S., Chaudhry G.M., Martin D.T., Neilan T.G., Mahmood S.S., Barac A., Groarke J.D., Hayek S.S. (2018). Ibrutinib-associated atrial fibrillation. JACC Clin. Electrophysiol..

[11] Barf T., Covey T., Izumi R., van de Kar B., Gulrajani M., van Lith B., van Hoek M., de Zwart E., Mittag D., Demont D. (2017). Acalabrutinib (ACP-196): A covalent Bruton tyrosine kinase inhibitor with a differentiated selectivity and in vivo potency profile. J. Pharmacol. Exp. Ther..

[12] Shadman M., Brown J.R., Mohseninejad L., Yang K., Burnett H., Neupane B., Williams R., Lamanna N., O’Brien S.M., Tedeschi A. (2025). Comparative efficacy of Bruton tyrosine kinase inhibitors in high-risk relapsed/refractory CLL: A network meta-analysis. Blood Adv..

